# A Review of Collagen Cross-Linking in Cornea and Sclera

**DOI:** 10.1155/2015/289467

**Published:** 2015-04-02

**Authors:** Xiao Zhang, Xiang-chen Tao, Jian Zhang, Zhi-wei Li, Yan-yun Xu, Yu-meng Wang, Chun-xiao Zhang, Guo-ying Mu

**Affiliations:** ^1^Department of Ophthalmology, Shandong Provincial Hospital, Shandong University, Jinan, Shandong 250000, China; ^2^Zibo Eye Hospital, No. 33-6, Songlingxi Street, Zichuan, Zibo, Shandong 255100, China

## Abstract

Riboflavin/UVA cross-linking is a technique introduced in the past decades for the treatment of keratoconus, keratectasia, and infectious keratitis. Its efficacy and safety have been investigated with clinical and laboratory studies since its first clinical application by Wollensak for the treatment of keratoconus. Although its complications are encountered during clinical practice, such as infection inducing risk, minimal invasion merits a further investigation on its future application in clinical practice. Recently, collagen cross-linking in sclera shows a promising prospect. In present study, we summarized the representative studies describing the clinical and laboratory application of collagen cross-linking published in past decades and provided our opinion on the positive and negative results of cross-linking in the treatment of ophthalmic disorders.

## 1. Introduction

Cross-linking is a natural phenomenon in cornea with aging in an enzymatic or nonenzymatic pattern [1] which will be accelerated in diabetes in a nonenzymatic pattern [2]. This suggests that artificial cross-linking may have similar effects to enhance tissue stiffness. Riboflavin/UVA corneal collagen cross-linking (often referred to as “CXL”) has been applied in the treatment of keratoconus in clinical practice in past decades. Besides its original application on keratoconus, CXL has been applied onto keratectasia and keratitis successfully. However, the mechanism underlying the therapeutic effect of CXL on corneal related disease is still not fully explored, and the efficacy of its clinical application needs to be further evaluated. Although riboflavin/blue light cross-linking still stays in animal experimental phase, it is promising to halt myopic progression and other sclera diseases since cross-linking can enhance sclera stiffness. In present study, we reviewed the clinical application and laboratory evidence reported in the past decades, by which to provide a more comprehensive scene of the collagen cross-linking application and assist our understanding on the efficacy and complications encountered during clinical and experimental practice.

## 2. Basic Research

Riboflavin/UVA corneal cross-linking is a process mediated by photooxidation between UVA (370 nm) and riboflavin (vitamin B_2_). In detail, UVA activates riboflavin into triplet, which in turn produces reactive oxygen species (ROS) including singlet oxygen. ROS reacts with collagen fibril molecules in cornea stroma and enhances the mechanical strength of cornea by forming new chemical bonds between amino/groups of collagen fibril molecules [3]. Besides, riboflavin also plays as a filter to reduce UVA penetrate cornea. Irradiation level of abrupt endothelial cytotoxicity closes to 0.36 mW/cm^2^ when illuminated at 370 nm wavelength without riboflavin [4, 5]. The riboflavin leads to a 50% increase in absorbance after 30 minutes of riboflavin treatment [6], with an absorbance coefficient of 56.36 ± 4.80/cm although other studies note a lower value [7]. According to this parameter, UVA irradiance density is 0.18 mW/cm^2^ for 370 nm wavelength at a depth of 400 *μ*m, which is less than half of the toxic level for endothelium. For this reason, the safety thickness of the cornea that can be treated by the standard protocol is 400 *μ*m.

Wollensak et al. [8] found in porcine corneas and thinner human corneas that the mechanical rigidity increased by 71.9% and 328.9%, respectively, and Young modulus increased by 80% and 450% after CXL with exposure time of 30 minutes and energy density of 3 mW/cm^2^. The stiffening of cornea is depth-dependent, which is being confined to approximately the anterior 200 *μ*m of the cornea [9, 10] and Kohlhaas et al. [9] believed 70% of UVA is absorbed within the anterior 200 *μ*m and 90% within the anterior 400 *μ*m.

Transmission electron microscope studies showed a 12.2% (3.96 nm) increase in collagen fibril diameters within this anterior region [11], demonstrating that collagen fibrils participate in the cross-linking process. However, X-ray scattering did not find the increase of fibril diameters in cross-linked corneas [12]. It is supposed that the cross-linked corneas may appear to have larger fibril diameters than untreated tissue observed by electron microscope, as the newly formed fibrils may increase resistance to the tissue shrinkage that is known to occur during tissue processing for electron microscopy [13].

Apoptosis of corneal stromal cells in the anterior corneal stroma can be observed at the early stage after CXL as evident by a stromal demarcation line appearing at a depth of approximately 300 *μ*m of cornea (epithelium-debrided) viewed under confocal microscopy [14], which may represent a boundary between cross-linked and non-cross-linked areas. However, cross-linked region is limited to a depth of 90–110 *μ*m in epithelium-intact corneas [15]. In vivo, confocal microscopy shows a loss of keratocytes in the anterior and mid stroma immediately after treatment. An increase of keratocytes can be observed at 3 months; the process of keratocytes repopulation is finalized at 6 months after CXL, while the damage of corneal endothelium is not observed during the process [16]. In keratoconus, collagen, proteoglycans, and keratocytes are abnormal [17–19]; however, it is still unknown what these migrating cells are doing in terms of collagen and proteoglycan deposition when they repopulate the stroma and to what extent a “normal” stromal ultra-structure is being attained.

## 3. Procedures and Modification

### 3.1. Standard Procedure

To date, the most commonly used protocol of CXL is “Dresden protocol” [20, 21]. After the application of topical anesthesia, the central epithelium with a diameter of 8-9 mm is removed with blade or laser. Then 0.1% riboflavin solution containing 20% dextran is dropped every 5 minutes for 30 minutes until the stroma is saturated with riboflavin, as evident by the chartreuse flare in the anterior chamber observed with blue light under slit-lamp. The cornea is then exposed to UVA (370 nm) for 30 minutes (energy density 3 mW/cm^2^). Riboflavin administration is continued during UVA illumination at the same intervals. Topical anesthesia is applied if necessary. After illumination, antibiotic eye drops and soft contact lens are applied on cornea to reduce pain and promote the growth of epithelium. The soft lens is removed until 5–7 days later when the healing of epithelium is observed. Application of topical antibiotics is required for 1 week after the operation and mild steroids may also be prescribed.

### 3.2. CXL with Hypoosmolar Riboflavin Solution

“Dresden protocol” [20, 21] is suitable for corneal thickness (CT) over 400 *μ*m. However, in many advanced keratoconus patients, CT is less than 400 *μ*m. Kymionis et al. [22] applied CXL with “Dresden protocol” [20, 21] to thin cornea (range 340 ± 399 *μ*m) and found a significant endothelial cell count loss. But no other signs of intraocular toxicity (lens-retina) were noted during the follow-up. Hafezi and associates [23] proposed using hypoosmolar riboflavin solution (0.1% riboflavin in 0.9% saline instead of dextran) as an alternative treatment protocol. But Hafezi et al. study did not include endothelial cell count measurements which could verify the safety of the technique. Gu et al. [24] investigated the safety and efficacy of using hypoosmolar riboflavin solution (0.1%) and UVA cross-linking for the treatment of keratoconus with the thin cornea (mean thinnest CT was 413.9 ± 12.4 *μ*m and 381.1 ± 7.3 *μ*m with and without epithelium). During a 12-month follow-up, the mean keratometric (*K*) value improved from 58.7 ± 3.5 diopters (D) to 57.7 ± 4.9 D (*P* = 0.611), and the endothelial cell density (ECD) was 2731.4 ± 191.8 cells/mm^2^ before treatment and decreased to 2722.5 ± 211.5 cells/mm^2^ (*P* = 0.208) at 6 months after treatment and returned to 2733.4 ± 222.6 cells/mm^2^ (*P* = 0.327) at 12 months. The ECD counts before and 6- and 12-month values after treatment showed no significant change (all with *P* > 0.05). Gu et al. proved that in patients with thin corneas (minimum CT less than 400 *μ*m after epithelial removal), hypoosmolar riboflavin solution should be an alternative protocol for CXL. However, Hafezi [25] reported a failure case by using hypoosmolar riboflavin solution in an extremely thin cornea (268 *μ*m without epithelial) and suggested that CXL could only be administrated in cases when stromal thickness is over 330 *μ*m before swelling with hypoosmolar riboflavin solution.

### 3.3. “Epithelium-On” CXL

Epithelial debridement in “Dresden protocol” [20, 21] may cause severe pain to the patient and may increase the risk of infection [26] and stromal haze [27]. Ophthalmologists attempted to find a modified CXL procedure without epithelium debridement, “epithelium-on” CXL procedure. “Epithelium-on” CXL was performed by applying an enhanced riboflavin solution with benzalkonium chloride [28], EDTA [29], and gentamicin [30] which can help riboflavin penetrating into corneal stroma through epithelium. Stojanovic et al. [31] compared the efficacy of CXL with and without epithelial removal for a 12-month follow-up and found that no significant difference between the two groups was observed at any point. Although there were a visual improvement and no progression after treatment, the effect of CXL was lower than previously reported for both groups. This discrepancy may be due to the fact that Stojanovic et al. used hypotonic 0.5% riboflavin instead of 0.1% riboflavin, which may lead to quicker oxygen consumption and therefore a reduction in the efficiency of CXL [31]. However, the effect of “epithelium-on” CXL varies from “less effective than standard CXL” [32] to “moderately effective” [33] to “appearing to halt keratoconus progression, with a statistically significant improvement in visual and topographic parameters” [34]. Though the result of “epithelium-on” CXL is variable, its noninvasive nature makes it potentially useful in special cases when epithelial debridement should be avoided, such as in patients with dry eyes, uncooperative patients, and patients with very thin corneas.

### 3.4. Iontophoresis Cross-Linking

As a modified procedure, transcorneal iontophoresis has been applied in corneal cross-linking (iontophoresis cross-linking, ionto-CXL). Iontophoresis is a noninvasive technique in which a weak electric current is used to enhance the penetration of electrically charged molecules into tissue. Its safety and efficacy of delivering drugs through cornea and into other ocular tissues had been proven [35, 36]. Riboflavin is suitable for the technique of iontophoresis due to small molecular weight, high solubility in water, and being negatively charged at physiological pH. Arboleda et al. [37] demonstrated that iontophoresis was a good candidate for riboflavin delivery into corneal stroma, but the formulation should be in a saline buffer instead of hyperosmotic dextran. The low molecular weight of the riboflavin-phosphate allowed for more rapid diffusion into stroma and could avoid corneal thinning due to hyperosmotic dextran [38]. Mencucci et al. [39] investigated the early modifications induced by ionto-CXL in ex vivo human corneas and found that morphological and biomolecular changes of corneas treated with ionto-CXL at 10 mW/cm^2^ for 9 minutes were similar to corneas treated with standard CXL at 3 mW/cm^2^ for 30 minutes. Bikbova and Bikbov [40] performed ionto-CXL on 22 eyes of 19 patients with progressive keratoconus. The current intensity was initially 0.2 mA and was gradually increased to 1.0 mA at an increment rate of 0.2 mA per 10 seconds to find out the individual tolerance and to avoid patients' discomfort. The total time that hypotonic riboflavin solution was administered by iontophoresis was 10 minutes. When examination showed complete impregnation with riboflavin for 10 minutes of iontophoresis, UVA (370 nm, 3 mW/cm^2^) was applied for 30 minutes, and during UVA exposure, hypotonic riboflavin drops were continued every 2 minutes. With a 6-month follow-up, the mean *K* values decreased by 2.3 D with visual acuity without statistically significant changes from preoperative values. No endothelial damage was observed during the follow-up, but the apoptotic keratocytes effect was only at 210–230 *μ*m depth while it was 270–300 *μ*m with standard CXL [14]. This less apoptotic effect might be due to reduced riboflavin supply during the UVA irradiation since the iontophoresis device had been removed and might also possibly lead to a lower biomechanical stiffening effect. Besides, the amount of riboflavin imbibed by iontophoresis was greater than conventional “epithelium-on” protocol, but less than standard “epithelium-off” protocol [37, 41]. What level of riboflavin concentration is needed in the stromal tissue to achieve a full cross-linking effect in treating keratoconus patients is unknown. Iontophoresis is a possible way of conveying more riboflavin in the corneal tissue with epithelium-on modality and reducing the administration time. Further studies are needed to demonstrate its long-term stabilization of cornea and unexpected side-effects.

### 3.5. Contact Lens-Assisted Cross-Linking

Although hypoosmolar riboflavin solution makes CXL on thin cornea possible, Wollensak et al. [42] stated that the unstable hypoosmolar riboflavin film used for thin corneas results in higher irradiance at the endothelial level than the dextran-riboflavin film, putting the endothelium at possible risk if the stroma is swollen to only 400 *μ*m. Besides, compared with normal cornea, the wide space of corneal collagen fibers of swollen cornea may induce weak effect of CXL [43]. Jacob et al. [44] started a new technique of contact lens-assisted collagen cross-linking (CACXL) in thin cornea (CT less than 400 *μ*m after epithelial removal). After epithelial abrasion, the isoosmolar riboflavin 0.1% in dextran was applied every 3 minutes for 30 minutes. An ultraviolet barrier-free soft contact lens (0.09 mm thickness, 14 mm diameter) soaked in isoosmolar riboflavin 0.1% for 30 minutes was placed on the cornea. Once the minimum corneal and contact lens thickness value is over 400 *μ*m, the UVA (370 nm) irradiance of 3 mW/cm^2^ was started in the precorneal and precontact lens region for 30 minutes, and isoosmolar riboflavin 0.1% was administrated at the same intervals. Intraoperative minimum CT changes were recorded with ultrasound pachymetry and optical coherence tomography (OCT). After illumination, the riboflavin-soaked contact lens was removed and a silicon hydrogel bandage contact lens was applied on the cornea until reepithelialization. In Jacob et al. study, 14 eyes diagnosed as keratectasia were included. Minimum CT was 377.2 ± 14.5 *μ*m after epithelial abrasion and 485.1 ± 15.8 *μ*m after contact lens wearing. Mean absolute increase CT along with the contact lens and precorneal riboflavin film was 107.9 ± 9.4 *μ*m. In follow-up from 6 to 7 months, corrected distance visual acuity (CDVA) had no significant change and maximum keratometry (*K*max) readings showed no progression. No significant endothelial loss was observed. In Jacob et al. study, corneas thickness was more than 350 *μ*m after epithelial abrasion, whether CACXL being suitable for extremely thin corneas is uncertain. Besides, the lesser patient population, short term follow-up, and absence of a control group (hypoosmolar CXL) are the limitations of Jacob et al. study.

### 3.6. Intrastromal Corneal Ring Segments and CXL

As a vision-correcting method, intrastromal corneal ring segments (ICRS) which were designed originally for the correction of mild to moderate myopia [45] are now being investigated to correct keratoconus without central corneal scarring. ICRS act by an arc-shortening effect and flatten the center of cornea as well as maintaining the existing biomechanical status in the underlying stroma [46]. In Coskunseven et al.'s [47] experience, the CT suitable for femtosecond Keraring ICRS implantation was at least 350 *μ*m at the thinnest corneal point and at least 450 *μ*m at the incision side. ICRS are effective in flattening the corneal shape and improving vision for keratoconus, but the effect of ICRS is variable depending on implantation techniques, types of ICRS, and stage of disease. The long-term effect of ICRS implantation should be concerned. In 13 keratoconus eyes [48], study with follow-up to 3 years, there was a significant decrease in average *K* values 6 months after ICRS, while 36 months after ICRS the *K* value increased to a level less than preoperative. The case indicated that cornea stabilization was not achieved by ICRS alone. CXL strengthens the biomechanical properties of cornea without significantly changing its shape. Combining two methods would be a logical solution to gain the benefits of both. Coskunseven et al. [49] compared the 2 sequence treatments ICRS implantation and CXL in patients with progressive keratoconus. With a mean follow-up of 7 months, group 2 (ICRS first and then CXL) achieved better outcome than group 1 (CXL first and then ICRS) with the increase of CDVA (3 lines versus 2 lines, *P* < 0.01) and decrease of manifest cylinder (2.48 D versus 1.76 D, *P* < 0.05). The differences between two groups in pachymetry, intraocular pressure (IOP), and endothelial cell count were also statistically significant (*P* < 0.05). There was no statistical difference between the two groups in uncorrected distance visual acuity (UDVA), spherical equivalent, or mean *K* (*P* > 0.05). The difference of two groups may be due to the stiffened cornea by CXL which decreases the flattening effect of ICRS implantation, thus restricting its effect and decreasing the maximum flattening potential. To achieve better effect, ICRS implantation should be performed first so the segments can reshape the cornea without restriction. Then perform CXL to further flatten the cornea and to enhance corneal biomechanics. The optimum interval between ICRS and CXL in combined treatment is uncertain, and ophthalmologists had different interval time [49–51]. Kl et al. [52] combined CXL and ICRS implantation with riboflavin injection into the corneal channel on the same day, demonstrating that riboflavin injection into the corneal channel for CXL treatment was safe and effective. Further studies can be planned to compare the effectiveness of different combinations.

### 3.7. Epithelial Island Cross-Linking

Though thin cornea could be swelling to safe thickness (over 400 *μ*m) with hypoosmolar riboflavin solution, the iatrogenic swelling effect was short acting, and the thinnest CT decreased significantly at the end of hypoosmolar riboflavin application [53], thus increasing the risk of corneal scarring and endothelial cells' damage. To solve this problem, a new technique that is epithelial island cross-linking (EI-CXL) with customized pachymetry-guided epithelial debridement was introduced [54]. In Mazzotta Cosimo and Ramovecchi study [54], EI-CXL technique was performed on 10 keratoconus patients with thin cornea (range 368–391 *μ*m). The epithelium in the paracentral and midperipheral cornea where the cornea is thicker was removed carefully and the thin apical cornea epithelium was preserved. 0.1% riboflavin and 20% dextran were administrated on cornea for 10 minutes according to Siena University protocol (Dresden modified) to avoid excessive dehydration [55]. CT must be at least 350 *μ*m and if not, hypotonic 0.1% riboflavin solution should be administrated for 10 minutes and recheck the thickness. Then illuminate cornea for 30 minutes at 3 mW/cm^2^. At the end of the procedure, soft contact lens was applied for 3 days. With a 12-month follow-up, corneal ectasia was stable with no significant improvement in UDVA, CDVA, and *K* value. ECD reduced by 2% with no statistical difference. Removing the epithelium where the cornea is thicker allows for a better penetration of riboflavin, ensuring a stronger biomechanical effect of CXL. The epithelial island protects the thin apical cornea from the UV radiation and its borders provide a refraction of UVA rays deviating their impact in an intermediate stromal level. The epithelial island together with an intrastromal riboflavin shield creates a safe barrier for corneal endothelium corresponding to the corneal thinnest point area. According to Mazzotta Cosimo and Ramovecchi study, the EI-CXL technique is safety and efficacy and reducing patients' discomfort; however, one month after EI-CXL, in vivo laser scanning confocal microscopy (IVCM) demonstrated keratocytes' apoptosis under the epithelial island at 150 *μ*m of depth on average (range 130–170 *μ*m) and in the paracentral and deepithelialized ring at 250 *μ*m depth on average (230–270 *μ*m), suggesting that the effect of cross-linking under epithelial island may be weaker. It still needs a larger cohort of patients and long-term follow-up to confirm the safety and efficacy of EI-CXL and evaluate whether it may become a standard treatment option for ectasia patient with thin cornea.

### 3.8. Orthokeratology and CXL

Orthokeratology uses specially designed and fitted rigid contact lenses to reshape the corneal contour to temporarily modify or eliminate refractive error, taking advantage of corneal plasticity [56]. Since orthokeratology can successful mold the cornea of normal eyes to correct myopia, it is valuable to investigate the effect of CXL to stabilize the outcome of corneal reshaping and improvement of visual acuity in patients with keratoconus after orthokeratology treatment. Nguyen and Chuck [57] selected 5 eyes from 4 keratoconus patients that underwent overnight orthokeratology for 3 months and then performed CXL following the Siena group protocol [55]. After a one-month gap for healing, overnight orthokeratology was resumed with piggy-back (rigid gas permeable contact lens (RGP) and silicone-hydrogel contact lenses) for three weeks and with RGP lenses only for two further months. All kinds of contact lenses were discontinued thereafter. In all cases, corneal topography showed improvement after orthokeratology; however, one month after orthokeratology interruption, corneal topography and corneal wavefront error returned at baseline level and remained stable at one-year follow-up. UDVA and best-spectacle corrected visual acuity (BSCVA) improved after orthokeratology; this improvement regressed one month after orthokeratology lens interruption (4 months after CXL) but did not return to baseline level. Only one eye showed an epithelial defect with asymptomatic iritis-like reaction and the complication resolved after a month of topical steroid therapy; no other relevant complications were noted one year after cross-linking. The study of Swarbrick showed that orthokeratology can reshape keratoconic corneas without significant complications, but the molding effect persists only for a short time compared to normal eyes [56]. Although CXL is safe and effective for keratoconus, it is not able to stabilize the orthokeratology molding effect. Interestingly, UCVA and BSCVA do seem to improve without full regression to baseline. Further studies with a larger cohort are needed to explain the outcomes and elucidate the efficacy of CXL in stabilizing orthokeratology.

## 4. Clinical Application

### 4.1. CXL in Keratoconus

Before the introduction of CXL, the treatment for keratoconus includes the wearing of spectacle glasses and contact lens, intrastromal corneal ring segment implantation [58], and keratoplasty [59]. However, all of these methods can only improve visual acuity but cannot arrest the progression of the disease. Cross-linking can increase the biomechanics of the cornea and thus stabilize the disorder. The first clinical application of CXL was conducted by Wollensak et al. for treating keratoconus under standard protocol [21]. He selected 22 patients (23 eyes) of keratoconus, with a mean follow-up of 23.2 ± 12.9 months, 65% of treated patients (15 patients) showed an improvement in best corrected visual acuity (BCVA) by an average of 1.26 lines, and 70% (16 patients) showed an improvement of mean *K* values by an average reduction of 2.01 D. Five patients showed stable *K* value, and only 1 patient had a minimal increase of the *K* value 0.28 D. In the fellow control eyes, however, 22% eyes (5 eyes) showed a progression of *K*max readings by an average of 1.48 D. The ECD and IOP had no significant change after cross-linking. The outcome of Wollensak et al. study was satisfying, and biomechanical stress-strain measurements showed that porcine and rabbit corneas had a significant increase in corneal rigidity after being treated by riboflavin/UVA cross-linking [60, 61]. After Wollensak et al. study, large numbers of clinic trials [62–64] were conducted and got satisfying results with *K*max reduction by approximately 2 D or more and modest visual acuity increase.

CXL only stabilized the cornea but could not improve patients' visual acuity greatly; thus CXL combined with photorefractive keratectomy (PRK) becomes an emerging treatment to improve patient's acuity. Kanellopoulos [65] divided 325 keratoconus eyes into two groups, in which 127 eyes underwent CXL followed by topography guided PRK 6 months later (sequential group), and 198 eyes underwent PRK and CXL on the same day (simultaneous group). The parameters for CXL are 0.1% riboflavin and UVA (365 to 375 nm) with energy density 3 mW/cm^2^ for 30 minutes. To ensure minimal tissue removal, the effective optical zone diameter was 5.5 mm (routine treatment diameter in PRK cases is no less than 6.5 mm) and transition zone was 1.5 mm. It is planned to treat about 70% of cylinder and whatever level of sphere on the prerequisite of no more than 50 *μ*m stromal removed. In follow-up from 24 to 68 months, there was a significant difference between two groups. In simultaneous group, mean UDVA (logMAR) improved from 0.96 ± 0.2 to 0.3 ± 0.2 and mean BSCVA (logMAR) from 0.39 ± 0.3 to 0.11 ± 0.16. Mean reduction in spherical equivalent refraction was 3.20 ± 1.40 D, mean haze score was 0.5 ± 0.3, and mean reduction in *K* was 3.50 ± 1.3 D and central CT decreased from 475 ± 55 *μ*m preoperatively to 405 ± 35 *μ*m. However, in sequential group, the mean UDVA improved from 0.9 ± 0.3 to 0.49 ± 0.25 and mean BSCVA from 0.41 ± 0.25 to 0.16 ± 0.22. Mean reduction in spherical equivalent refraction was 2.50 ± 1.20 D, mean haze score was 1.2 ± 0.5, and mean reduction in *K* was 2.75 ± 1.30 D; mean central CT decreased from 465 ± 45 *μ*m preoperatively to 395 ± 25 *μ*m. *t*-test revealed that the simultaneous group had better outcome of BSCVA (*P* < 0.001), spherical equivalent reduction (*P* < 0.005), mean *K* reduction (*P* < 0.005), and corneal haze score (*P* < 0.002) at last follow-up. ECD preoperatively and at final follow-up was unchanged (*P* > 0.05) in both groups. Simultaneous group seemed to be an “enhanced” CXL compared to sequential group, either due to better penetration of riboflavin through the ablated stroma or due to the absence of Bowman's layer through PRK. Besides, compared with sequential group, simultaneous group had three advantages: (1) the combination reduces patient's treating time, (2) combining two procedures at one time minimizes the potential superficial stromal scarring, and (3) when PRK is performed after the CXL procedure, some of the cross-linked anterior cornea will be removed, minimizing the effectiveness of CXL. Thus a combination procedure of CXL immediately after topography guided PRK appears to be a better way for the visual rehabilitation in progressing keratoconus.

However, with the progression of keratoconus, CT becomes thinner and even less than 400 *μ*m; thus PRK cannot completely correct the refraction in patients with high refractive error. Recently, ophthalmologists found that phakic intraocular lens (IOL) is suitable for patients with moderate to severe ametropia [66, 67]. Fadlallah et al. [68] implanted implantable collamer lens (ICL) in 16 high myopic eyes of keratoconus with stable refraction after CXL. CXL was performed by “Dresden protocol” [20, 21], and ICL was implanted 6 months after CXL when the cornea was stable. One week before ICL implantation, laser iridotomy was performed. ICL was inserted in the posterior chamber through a 3.2 mm corneal tunnel incision. Six months after ICL implantation, spherical equivalent decreased from −7.24 ± 3.53 to −0.89 ± 0.76 D (*P* = 0.001) and mean cylinder decreased from 2.64 ± 1.28 to 1.16 ± 0.64 D (*P* = 0.001). Mean *K*max decreased from 52.29 ± 4.79 D at baseline to 51.33 ± 4.41 D at 6 months after CXL (*P* = 0.001) and to 50.49 ± 4.07 D at 6 months after ICL implantation (*P* = 0.001). In Fadlallah et al.'s study, ICL implanted after 6 months with *K* values were flatted or at least remained stable; continuous flattening of *K* values was also observed after ICL implantation, but that did not affect visual acuity after ICL implantation. IOP increased in most patients in the first week after ICL implantation and was successfully managed with topical IOP-lowering drops. No other severe intra- or posttreatment complications were noted in any of the cases. The study demonstrates that the implantation of ICL following CXL is an acceptable treatment for patients with high refractive error in keratoconus. However, the “quality” of vision such as occurrence of glares and halos was not mentioned in Fadlallah et al.'s study. Besides, it is better to implant ICL when cornea is stable to ensure the outcome is more reliable.

Keratoconus is a noninflammatory degeneration of cornea characterized by progression of corneal protrusion and stromal thinning with visual loss. It has been demonstrated that the number of diagonal links of collagen fibrils is significantly reduced in keratoconus [69] and thus weakens the mechanical stability of cornea. CXL can enhance the mechanical strength of cornea by forming new chemical bonds [3] and halt the progression of the disease. For patients with slight to moderate refractive error, simultaneous PRK combined with CXL may be an alternative method. For advanced keratoconus patients with high refractive error, implant ICL after CXL can not only stabilize the cornea but also improve visual acuity greatly.

### 4.2. CXL in Keratectasia

Keratectasia which is also called iatrogenic ectasia that happened after laser surgery, such as laser-assisted in situ keratomileusis (LASIK) and PRK, was first reported by Seiler et al. [70] in 1998. It was defined as topographic steepening of 5 D or more compared with immediate postoperative appearance, loss of 2 lines or more of Snellen acuity, and a change in manifest refraction of 2 D or more of either sphere or cylinder. The occurrence rate of keratectasia ranges from 0.04% to 0.6% after LASIK [71–73] and less after PRK [74]. CXL can strengthen cornea by forming new chemical bonds [3]; thus it is efficacy in the arrest of keratectasia theoretically. Richoz et al. [75] administrated CXL to 26 eyes (26 patients) with progressive keratectasia after LASIK (23 eyes) and PRK (3 eyes). CXL was performed with “Dresden protocol” [20, 21] except for 8 patients with central CT less than 400 *μ*m, in whom hypoosmolar riboflavin solution was used before cross-linking. After central 8.0 mm of corneal epithelium was debrided, 0.1% riboflavin was instilled every 3 minutes for 30 minutes; UVA irradiation (energy density 3 mW/cm^2^) was applied for 30 minutes. During illumination, riboflavin was applied every 5 minutes. With a follow-up of 12–62 months, mean CDVA (logMAR) was 0.5 ± 0.3 before CXL and improved to 0.3 ± 0.14 after treatment. CDVA improved in 19 patients and remained stable in 7. No patient showed deterioration. Mean *K*max before CXL was 52.8 ± 5 D and improved to 50.9 ± 4.9 D; the improvement was 1.9 ± 1.9 D. Mean central CT was 9 ± 17 *μ*m thinner after CXL. All patients showed no signs of endothelial damage. Richoz et al. study implied that CXL could arrest the progression of keratectasia and improve CDVA and decrease *K*max, while in a study by Vinciguerra et al. [76] there were no significant topographic changes (average keratometry, flat keratometry, or steep keratometry) in keratectasia after CXL. This suggested that unlike keratoconic corneas ectatic corneas may have a less robust response to CXL. Although the cause for this potential difference is unsure, several explanations have been suggested. One is that CXL mainly strengthens the anterior stroma, including the LASIK flap, which contributes little to the mechanical stability of the cornea. The riboflavin penetration may be reduced in corneas that have undergone laser surgery, thus affecting the CXL result. Besides, differences in the pathophysiologic features of keratoconus and keratectasia may also affect the outcome of CXL [77, 78].

Aiming to correct the refractive error in patients with keratectasia, Kanellopoulos and Binder [79] performed PRK combined with CXL in 32 eyes of 22 patients with keratectasia which happened 1 to 4 years after LASIK. Due to the light flattening effect of CXL, Kanellopoulos and Binder planned to correct 70% of cylinder and sphere, thus ensuring no more than 50 *μ*m of stromal was removed to avoid exacerbation of keratectasia. A phototherapeutic keratectomy (PTK) (6.5 mm diameter and 50 *μ*m depth) to remove the corneal epithelium and partial topography guided PRK was performed to correct visual acuity. 0.02% mitomycin C solution was applied on the ablated cornea bed for 20 seconds. CXL was performed as “Dresden protocol” [20, 21] 10 minutes after laser surgery. With a follow-up range from 6 to 59 months, UDVA improved in 27 eyes, was unchanged in 4 eyes, and worsened in 1 eye. Mean refractive error decreased by more than 2.50 D in 27 of 32 eyes, remained stable in 2 eyes, and increased by 0.75 D in 3 eyes. Simultaneous topography guided PRK and CXL appear to be effective in the treatment of keratectasia. The procedure is easy to perform, but more researches are needed to determine the accurate laser ablation and refraction correction since the flattening effect of CXL is not uniform in all keratectasia.

The residual corneal stromal bed less than 250 *μ*m could induce keratectasia and corneal flap plays little biomechanical effect [80]. We proposed a PTK-PRK-CXL method, that is, to perform PRK on flap after epithelium is removed with PTK, and then perform CXL with “Dresden protocol” [20, 21]. We selected 6 keratectasia patients (8 eyes); the mean corneal flap thickness is 138 mm ± 26 (SD) monitored by OCT. We used PTK (8.5 mm diameter and 50 *μ*m depth) to remove epithelium. Then PRK is performed on flap to correct refractive error. In follow-up at 6 months, results showed that mean UDVA (LogMAR) improved from 0.775 ± 0.675 to 0.437 ± 0.459 (*P* < 0.05) and mean CDVA (LogMAR) improved from 0.387 ± 0.36 to 0.325 ± 0.319 (*P* > 0.05). Steep *K* values and flat *K* values had a significant reduction (both *P* < 0.05). During the follow-up, ECD and IOP had no significant change. In our experience, the PRK was performed only on the corneal flap during CXL-PRK combination treatment. Because cornea stability of keratectasia is mainly affected by the stroma beneath cornea flap, the combination of CXL and PRK reserved the intact of the corneal stroma beneath the corneal flap by which impedes the progression of keratectasia. However, in keratectasia patients with high refractive error, PRK only performed above cornea flap could not acquire satisfying outcome. We assume that it is feasible to combine CXL with ICL implantation to keratectasia with high refractive error. This assumption still needs more efforts to determine its safety and efficacy before clinical application.

Corneal biomechanics of keratectasia is artificially weakened during refractive surgery after removing tissue from the stromal bed. Clinical trials have been proved that CXL should be the first choice for patients with progressing keratectasia. CXL combined with partial PRK/PTR-PRK on cornea flap can correct part of refractive error. In terms of high refractive error, further researches are needed to explore new treating method.

### 4.3. CXL in Infectious Keratitis

The commonly encountered pathogens of infectious keratitis include bacteria, fungi, virus, and* Acanthamoeba*. Trauma, contact lens wearing, and ocular surface diseases [81–85] are risk factors for the development of keratitis. Traditional therapy includes topical and/or systemic antimicrobial drugs application. In less effective cases or severe keratitis, corneal transplantation may be needed. Researches had demonstrated the antimicroorganism effect of UV rays [86, 87] and riboflavin could act as a photomediator to inactivate pathogens in plasma, platelets, and red blood cells [88, 89]. Spoerl et al. [90] performed CXL onto porcine eyes and exposed the corneas to trypsin, pepsin, and collagenase solutions and found that CXL treated corneas dissolved on days 5, 13, and 14 after exposure, respectively, while corresponding control corneas dissolved on days 2, 6, and 6. The study demonstrated that CXL could increase the resistance of the cornea against enzymatic digestion. Besides, reactive oxygen species produced by CXL can eliminate or suppress the proliferation of pathogens through destroying the nucleic acids of pathogens [73, 90]. Amount of in vitro studies has shed light on the location of CXL in keratitis [91, 92]. In recent years, several in vivo studies have achieved satisfying results. The primary outcome was the healing of corneal ulcer, defined as reepithelialization, block of corneal melting, and visual acuity recovery. Ferrari et al. [93] cured 1 eye with* Escherichia coli* derived keratitis by CXL. Li et al. [94] cured 8 eyes with fugal keratitis. Müller et al. [95] perform CXL onto 6 eyes with corneal melting induced by variable pathogens including bacteria, fungi, and* Acanthamoeba*, in which the ulcer in 4 eyes was healed and other 2 eyes were healed after applying additional surgical procedures. Arance-Gil et al. [96] performed CXL on one orthokeratology patient with* Acanthamoeba* keratitis. The patient was diagnosed by confocal microscopy. Corneal condition worsened after aggressive medication application for 1 year and CXL was performed. The symptoms and corneal appearance improved significantly after CXL and* Acanthamoeba* treatment was interrupted 3 months later with no cysts observed by confocal microscopy. To resolve the deficient corneal reepithelization, amniotic membrane implantation was performed 6 months after CXL. Corneal melting and glaucoma were observed 8 months after CXL, glaucoma and cataract surgeries, and penetrating keratoplasty were performed with no signs of rejection or other complications and the BCVA reached 20/60. In this case, CXL was effective to eliminate* Acanthamoeba* active and cystic forms. However, the outcomes of CXL on* Acanthamoeba* were different [97, 98]. This may be explained by the fact that the effectiveness depth of CXL limited to the anterior 300 *μ*m [14] and the treatment might be unsuccessful when microorganisms are penetrating too deep. These findings support that CXL could be an acceptable complementary method for the treatment of infectious keratitis. However, all of these studies are combined with antibiotic drugs and the effect of cross-linking alone is uncertain. It is found that the success rate is higher for bacterial infections than fungal infections and that cross-linking should not be performed in eyes with prior herpes simplex [13]. However, in vitro studies performed by Makdoumi et al. [99] and Martins et al. [100] implied that the parameters used in treatment of keratoconus might not fit for cure of keratitis. It seems that antimicrobial effect of cross-linking will be enhanced when dose of UVA is increased. At present, CXL should be applied to cases of severe nonresponsiveness to antimicrobial drugs for keratitis before undertaking emergency keratoplasty [101]. More efforts need to be done to determine the best parameters and indications of cross-linking in keratitis.

## 5. Experimental Study

### 5.1. CXL in Sclera

Compared with normal eye, the thickness of sclera is thinner in myopia eyes, especially at the posterior pole [102], and the biomechanical parameters are weaker [103]. The thinning and stiffness weakening of sclera are a common pathologic change in myopias [103]. Thus strengthening the posterior sclera is expected to halt myopic progression and decrease visual loss. The sclera of porcine is permeable to compounds with a molecular weight up to 120 kDa [104], while the permeability of human and rabbit sclera can reach up to 150 kDa [105, 106]; thus riboflavin (456 Da) can penetrate into the sclera theoretically. Wollensak et al. [107] proved CXL increased the stiffness of porcine and human sclera; however, Wollensak and Spoerl [108] also found retinal damage in the rabbit eyes when exposed to 0.1% riboflavin and 365 nm wavelength at 4.2 mW/cm^2^ for 30 minutes although an increased ultimate stress up to 228% was measured. Zhang et al. [109] irradiated rabbit sclera with different time and found that the optimal UVA irradiation time for rabbit sclera is 40 minutes, under the condition of UVA wavelength 365 nm (energy density 3 mW/cm^2^). With such parameters, the ultimate stress, Young modulus, and the physiological modulus had a significant increase without retinal damages. However, Zhang et al. study had two limitations: the irradiation area includes equatorial sclera and parts of posterior sclera, and the eyeball is spherical which led to irradiation being uneven. The permeability and structure of sclera of human, rabbit, and porcine are different [104, 110]; thus the outcomes may be different due to different sclera model. Zhang et al. [111] compared the biomechanical properties of human, porcine, and rabbit sclera before and after cross-linking and found that under the same condition (0.1% riboflavin with UVA 365 nm for 40 minutes with energy density 3 mW/cm^2^ at a distance of 5 cm from the scleral plane) cross-linking increases the biomechanical stiffness of rabbit sclera but not human or porcine sclera, and in terms of stress-strain biomechanical studies, porcine sclera was closer to human sclera compared with rabbit sclera. Thus, porcine sclera is a better study model for sclera collagen cross-linking.

### 5.2. Riboflavin/Blue Light Cross-Linking

UVA has been widely used in corneal collagen cross-linking. However, cross-linking on sclera is only stayed in pilot phase. The main limitation is UVA penetration ability in sclera. 445 nm (blue light) is another absorption peak of riboflavin besides 365 nm (UVA) and it is also effective in cross-linking with riboflavin [112]. Theoretically, blue light has better penetration ability in sclera compared to UVA considering the negative relation between wavelength and penetrating depth in tissue. Besides, compared with 365 nm, the longer the wavelength the lower the potential for biological damage [113]. Iseli et al. [114] performed blue light (465 nm at 26 mW/cm^2^) cross-linking on 6 rabbits sclera for 20 minutes. Four weeks later, compared to the untreated control eye, the stress-strain measurement showed a threefold increase of sclera stiffness in the eyes with sclera cross-linking. Histological examination proved that the retinal cells and pigment epithelium were normal and anatomically undamaged of treated eyes. In contrast to Wollensak et al. [108], Iseli et al. [114] administered riboflavin for 20 minutes (Wollensak et al. [108] only administered riboflavin for 5 minutes) prior to the blue light irradiation to achieve deeper penetration of riboflavin and hence achieved better shielding of light and cytotoxic exposure to the retina. Riboflavin/blue light cross-linking on sclera is a promising approach for stiffening the sclera while minimizing the retinal damage. Further investigations concerning the retinal damage threshold and the best parameters of riboflavin/blue light cross-linking are needed.

## 6. Energy Modulation System

Wavelength of 365 nm and 430 nm is absorption peak of riboflavin. According to the formulation of *W* = *h* × *c*/*λ* (*λ* = wavelength, *c* = velocity of light, and *h* = Planck's constant) [61], radiation with 365 nm achieves a greater CXL effect; thus 365 nm was chosen for the CXL. Biomechanical effect studies with an irradiance of 3 mW/cm^2^ for 5 to 60 minutes found that a significant biomechanical increase in stiffness began at 15 minutes and did not increase over 45 minutes, so it is concluded that the optimal irradiation time was 30 minutes [115]. Now, there are various CXL machines like CCL-365 (PESCHKE Meditrade GmbH, Huenenberg, Switzerland) and UV-X (IROC GmbH, Zurich, Switzerland), which use UV light emission diodes supplying a homogeneous irradiation density of 3 mW/cm^2^ on a circular area of 8 mm on cornea. The choice of an 8 mm in diameter guarantees UVA irradiation only on the central cornea instead of limbus, sclera, or the goblet cells. Some devices make it possible to choose the size of the area to be irradiated, making CXL possible for other diseases, such as keratitis. The high degree of homogeneity of the radiation system can prevent local radiation peaks (“hot spots” at individual diodes), which could cause local damage to the endothelium. In accordance with Bunsen-Roscoe law, one achieves the same photochemical effect with a reduced irradiation time and a corresponding increased irradiation intensity, while total dose remains the same. Such devices have been offered from companies such as Avedro, Inc. (KXL, Waltham, MA, USA). Avedro system advanced the science and technology of CXL and refractive correction, from treating corneal pathology with UV-X devices, to developing noninvasive refractive procedures with advanced KXL II System.

## 7. Conclusion

Riboflavin/UVA cross-linking has been revealed as an effective and minimally invasive intervention for the treatment of keratoconus, keratectasia, and infectious keratitis. Since the first study introduced decades ago, many modifications and improvements to the original protocol have been carried out and received satisfying outcomes. Animal experiments also proved the prospect of sclera cross-linking in the treatment of pathological myopia. However, more studies are needed to elaborate some questions encountered during the clinical or laboratory application, including the long-term efficacy and safety of cross-linking, the combination modality of cross-linking and keratoplasty surgery, the outcomes after cross-linking twice or more times, and the efficacy and complications of cross-linking with longer wavelength light.

## References

[1] Elsheikh A., Wang D., Brown M., Rama P., Campanelli M., Pye D. (2007). Assessment of corneal biomechanical properties and their variation with age. *Current Eye Research*.

[2] Sady C., Khosrof S., Nagaraj R. (1995). Advanced Maillard reaction and crosslinking of corneal collagen in diabetes. *Biochemical and Biophysical Research Communications*.

[3] Raiskup F., Spoerl E. (2013). Corneal crosslinking with riboflavin and ultraviolet A. I. principles. *The Ocular Surface*.

[4] Wollensak G., Spoerl E., Wilsch M., Seiler T. (2003). Endothelial cell damage after riboflavin-ultraviolet—a treatment in the rabbit. *Journal of Cataract and Refractive Surgery*.

[5] Mazzotta C., Traversi C., Baiocchi S. (2008). Corneal healing after riboflavin ultraviolet-a collagen cross-linking determined by confocal laser scanning microscopy in vivo: early and late modifications. *The American Journal of Ophthalmology*.

[6] Wollensak G., Aurich H., Wirbelauer C., Sel S. (2010). Significance of the riboflavin film in corneal collagen crosslinking. *Journal of Cataract & Refractive Surgery*.

[7] Koppen C., Gobin L., Tassignon M.-J. (2010). The absorption characteristics of the human cornea in ultraviolet-a crosslinking. *Eye & Contact Lens*.

[8] Wollensak G., Spoerl E., Seiler T. (2003). Stress-strain measurements of human and porcine corneas after riboflavin-ultraviolet-A-induced cross-linking. *Journal of Cataract and Refractive Surgery*.

[9] Kohlhaas M., Spoerl E., Schilde T., Unger G., Wittig C., Pillunat L. E. (2006). Biomechanical evidence of the distribution of cross-links in corneastreated with riboflavin and ultraviolet a light. *Journal of Cataract and Refractive Surgery*.

[10] Søndergaard A. P., Hjortdal J., Breitenbach T., Ivarsen A. (2010). Corneal distribution of riboflavin prior to collagen cross-linking. *Current Eye Research*.

[11] Wollensak G., Wilsch M., Spoerl E., Seiler T. (2004). Collagen fiber diameter in the rabbit cornea after collagen crosslinking by riboflavin/UVA. *Cornea*.

[12] Hayes S., Kamma-Lorger C. S., Boote C. (2013). The effect of riboflavin/UVA collagen cross-linking therapy on the structure and hydrodynamic behaviour of the ungulate and rabbit corneal stroma. *PLoS ONE*.

[13] Meek K. M., Hayes S. (2013). Corneal cross-linking—a review. *Ophthalmic & Physiological Optics*.

[14] Seiler T., Hafezi F. (2006). Corneal cross-linking-induced stromal demarcation line. *Cornea*.

[15] Filippello M., Stagni E., O'Brart D. (2012). Transepithelial corneal collagen crosslinking: bilateral study. *Journal of Cataract & Refractive Surgery*.

[16] Mazzotta C., Balestrazzi A., Traversi C. (2007). Treatment of progressive keratoconus by riboflavin-UVA-induced cross-linking of corneal collagen: ultrastructural analysis by Heidelberg retinal tomograph II in vivo confocal microscopy in humans. *Cornea*.

[17] Funderburgh J. L., Panjwani N., Conrad G. W., Baum J. (1989). Altered keratan sulfate epitopes in keratoconus. *Investigative Ophthalmology and Visual Science*.

[18] Meek K. M., Tuft S. J., Huang Y. (2005). Changes in collagen orientation and distribution in keratoconus corneas. *Investigative Ophthalmology and Visual Science*.

[19] Buddecke E., Wollensak J. (1966). Acid mucopolysaccharides and glycoproteins in the human cornea in relationship to age and keratoconus. *Albrecht von Graefe's Archive for Clinical and Experimental Ophthalmology*.

[20] Kymionis G. D., Mikropoulos D. G., Portaliou D. M., Voudouragkaki I. C., Kozobolis V. P., Konstas A. G. P. (2013). An overview of corneal collagen cross-linking (CXL). *Advances in Therapy*.

[21] Wollensak G., Spoerl E., Seiler T. (2003). Riboflavin/ultraviolet-A-induced collagen crosslinking for the treatment of keratoconus. *American Journal of Ophthalmology*.

[22] Kymionis G. D., Portaliou D. M., Diakonis V. F., Kounis G. A., Panagopoulou S. I., Grentzelos M. A. (2012). Corneal collagen cross-linking with riboflavin and ultraviolet-a irradiation in patients with thin corneas. *American Journal of Ophthalmology*.

[23] Hafezi F., Mrochen M., Iseli H. P., Seiler T. (2009). Collagen crosslinking with ultraviolet-A and hypoosmolar riboflavin solution in thin corneas. *Journal of Cataract & Refractive Surgery*.

[24] Gu S., Fan Z., Wang L., Tao X., Zhang Y., Mu G. (2014). Corneal collagen cross-linking with hypoosmolar riboflavin solution in keratoconic corneas. *BioMed Research International*.

[25] Hafezi F. (2011). Limitation of collagen cross-Linking with hypoosmolar riboflavin solution: failure in an extremely thin cornea. *Cornea*.

[26] Sharma N., Maharana P., Singh G., Titiyal J. S. (2010). *Pseudomonas* keratitis after collagen crosslinking for keratoconus: case report and review of literature. *Journal of Cataract & Refractive Surgery*.

[27] Mazzotta C., Balestrazzi A., Baiocchi S., Traversi C., Caporossi A. (2007). Stromal haze after combined riboflavin-UVA corneal collagen cross-linking in keratoconus: in vivo confocal microscopic evaluation. *Clinical and Experimental Ophthalmology*.

[28] Kissner A., Spoerl E., Jung R., Spekl K., Pillunat L. E., Raiskup F. (2010). Pharmacological modification of the epithelial permeability by benzalkonium chloride in UVA/Riboflavin corneal collagen cross-linking. *Current Eye Research*.

[29] Nakamura T., Yamada M., Teshima M. (2007). Electrophysiological characterization of tight junctional pathway of rabbit cornea treated with ophthalmic ingredients. *Biological & Pharmaceutical Bulletin*.

[30] Chang S.-W., Chi R.-F., Wu C.-C., Su M.-J. (2000). Benzalkonium chloride and gentamicin cause a leak in corneal epithelial cell membrane. *Experimental Eye Research*.

[31] Stojanovic A., Zhou W., Utheim T. P. (2014). Corneal collagen cross-linking with and without epithelial removal: a contralateral study with 0.5% hypotonic riboflavin solution. *BioMed Research International*.

[32] Koppen C., Wouters K., Mathysen D., Rozema J., Tassignon M.-J. (2012). Refractive and topographic results of benzalkonium chloride-assisted transepithelial crosslinking. *Journal of Cataract and Refractive Surgery*.

[33] Spadea L., Mencucci R. (2012). Transepithelial corneal collagen cross-linking in ultrathin keratoconic corneas. *Clinical Ophthalmology*.

[34] Filippello M., Stagni E., O'Brart D. (2012). Transepithelial corneal collagen crosslinking: bilateral study. *Journal of Cataract and Refractive Surgery*.

[35] Yoo S. H., Dursun D., Dubovy S. (2002). Iontophoresis for the treatment of paecilomyces keratitis. *Cornea*.

[36] Sarraf D., Lee D. A. (1994). The role of iontophoresis in ocular drug delivery. *Journal of Ocular Pharmacology*.

[37] Arboleda A., Kowalczuk L., Savoldelli M. (2014). Evaluating in vivo delivery of riboflavin with Coulomb-controlled iontophoresis for corneal collagen cross-linking: a pilot study. *Investigative Ophthalmology & Visual Science*.

[38] Holopainen J. M., Krootila K. (2011). Transient corneal thinning in eyes undergoing corneal cross-linking. *American Journal of Ophthalmology*.

[39] Mencucci R., Ambrosini S., Paladini I. (2015). Early effects of corneal collagen cross-linking by iontophoresis in ex vivo human corneas. *Graefe's Archive for Clinical and Experimental Ophthalmology*.

[40] Bikbova G., Bikbov M. (2014). Transepithelial corneal collagen cross-linking by iontophoresis of riboflavin. *Acta Ophthalmologica*.

[41] Mastropasqua L., Nubile M., Calienno R. (2014). Corneal cross-linking: intrastromal riboflavin concentration in iontophoresis-assisted imbibition versus traditional and transepithelial techniques. *The American Journal of Ophthalmology*.

[42] Wollensak G., Spoerl E., Reber F., Seiler T. (2004). Keratocyte cytotoxicity of riboflavin/UVA-treatment in vitro. *Eye*.

[43] Creech J. L., Do L. T., Fatt I., Radke C. J. (1998). In vivo tear-film thickness determination and implications for tear-film stability. *Current Eye Research*.

[44] Jacob S., Kumar D. A., Agarwal A., Basu S., Sinha P. (2014). Contact lens-assisted collagen cross-linking (CACXL): a new technique for cross-linking thin corneas. *Journal of Refractive Surgery*.

[45] Ruckhofer J., Twa M. D., Schanzlin D. J. (2000). Clinical characteristics of lamellar channel deposits after implantation of Intacs. *Journal of Cataract and Refractive Surgery*.

[46] Burris T. E., Ayer C. T., Evensen D. A., Davenport J. M. (1991). Effects of intrastromal corneal ring size and thickness on corneal flattening in human eyes. *Refractive & Corneal Surgery*.

[47] Coskunseven E., Kymionis G. D., Tsiklis N. S. (2008). One-year results of intrastromal corneal ring segment implantation (KeraRing) using femtosecond laser in patients with keratoconus. *American Journal of Ophthalmology*.

[48] Alió J. L., Shabayek M. H., Artola A. (2006). Intracorneal ring segments for keratoconus correction: long-term follow-up. *Journal of Cataract and Refractive Surgery*.

[49] Coskunseven E., Jankov M. R., Hafezi F., Atun S., Arslan E., Kymionis G. D. (2009). Effect of treatment sequence in combined intrastromal corneal rings and corneal collagen crosslinking for keratoconus. *Journal of Cataract and Refractive Surgery*.

[50] Ertan A., Karacal H., Kamburoglu G. (2009). Refractive and topographic results of transepithelial cross-linking treatment in eyes with intacs. *Cornea*.

[51] Saelens I. E. Y., Bartels M. C., Bleyen I., Van Rij G. (2011). Refractive, topographic, and visual outcomes of same-day corneal cross-linking with ferrara intracorneal ring segments in patients with progressive keratoconus. *Cornea*.

[52] Kl A., Kamburoglu G., Aknc A. (2012). Riboflavin injection into the corneal channel for combined collagen crosslinking and intrastromal corneal ring segment implantation. *Journal of Cataract and Refractive Surgery*.

[53] Kaya V., Utine C. A., Ylmaz Ö. F. (2012). Intraoperative corneal thickness measurements during corneal collagen cross-linking with hypoosmolar riboflavin solution in thin corneas. *Cornea*.

[54] Mazzotta Cosimo C., Ramovecchi V. (2014). Customized epithelial debridement for thin ectatic corneas undergoing corneal cross-linking: epithelial island cross-linking technique. *Clinical Ophthalmology*.

[55] Caporossi A., Mazzotta C., Baiocchi S., Caporossi T. (2010). Long-term results of riboflavin ultraviolet a corneal collagen cross-linking for keratoconus in Italy: the Siena eye cross study. *American Journal of Ophthalmology*.

[56] Swarbrick H. A. (2006). Orthokeratology review and update. *Clinical and Experimental Optometry*.

[57] Nguyen M. K., Chuck R. S. (2013). Corneal collagen cross-linking in the stabilization of PRK, LASIK, thermal keratoplasty, and orthokeratology. *Current Opinion in Ophthalmology*.

[58] Siganos C. S., Kymionis G. D., Kartakis N., Theodorakis M. A., Astyrakakis N., Pallikaris I. G. (2003). Management of keratoconus with Intacs. *American Journal of Ophthalmology*.

[59] Frost N. A., Wu J., Lai T. F., Coster D. J. (2006). A review of randomized controlled trials of penetrating keratoplasty techniques. *Ophthalmology*.

[60] Spörl E., Schreiber J., Hellmund K., Seiler T., Knuschke P. (2000). Cross-linking effects in the cornea of rabbits. *Ophthalmologe*.

[61] Spoerl E., Huhle M., Seiler T. (1998). Induction of cross-links in corneal tissue. *Experimental Eye Research*.

[62] Raiskup-Wolf F., Hoyer A., Spoerl E., Pillunat L. E. (2008). Collagen crosslinking with riboflavin and ultraviolet-A light in keratoconus: long-term results. *Journal of Cataract & Refractive Surgery*.

[63] Jankov M. R., Hafezi F., Beko M. (2008). Corneal Cross-linking for the treatment of keratoconus: preliminary results. *Arquivos Brasileiros de Oftalmologia*.

[64] El-Raggal T. M. (2009). Riboflavin-ultraviolet a corneal cross-linking for keratoconus. *Middle East African Journal of Ophthalmology*.

[65] Kanellopoulos A. J. (2009). Comparison of sequential vs same-day simultaneous collagen cross-linking and topography-guided PRK for treatment of keratoconus. *Journal of Refractive Surgery*.

[66] Kymionis G. D., Grentzelos M. A., Karavitaki A. E., Zotta P., Yoo S. H., Pallikaris I. G. (2011). Combined corneal collagen cross-linking and posterior chamber toric implantable collamer lens implantation for keratoconus. *Ophthalmic Surgery, Lasers & Imaging*.

[67] Güell J. L., Morral M., Malecaze F., Gris O., Elies D., Manero F. (2012). Collagen crosslinking and toric iris-claw phakic intraocular lens for myopic astigmatism in progressive mild to moderate keratoconus. *Journal of Cataract and Refractive Surgery*.

[68] Fadlallah A., Dirani A., El Rami H., Cherfane G., Jarade E. (2013). Safety and visual outcome of visian toric ICL implantation after corneal collagen cross-linking in keratoconus. *Journal of Refractive Surgery*.

[69] Sherwin T., Brookes N. H. (2004). Morphological changes in keratoconus: pathology or pathogenesis. *Clinical & Experimental Ophthalmology*.

[70] Seiler T., Koufala K., Richter G. (1998). Iatrogenic keratectasia after laser in situ keratomileusis. *Journal of Refractive Surgery*.

[71] Rad A. S., Jabbarvand M., Saifi N. (2004). Progressive keratectasia after laser in situ keratomileusis. *Journal of Refractive Surgery*.

[72] Ferreira T. B., Marques E. F., Filipe H. P. (2014). Combined corneal collagen crosslinking and secondary intraocular lens implantation for keratectasia after radial keratotomy. *Journal of Cataract & Refractive Surgery*.

[73] Raiskup F., Spoerl E. (2013). Corneal crosslinking with riboflavin and ultraviolet A. Part II. clinical indications and results. *The Ocular Surface*.

[74] Hamilton D. R., Johnson R. D., Lee N., Bourla N. (2008). Differences in the corneal biomechanical effects of surface ablation compared with laser in situ keratomileusis using a microkeratome or femtosecond laser. *Journal of Cataract and Refractive Surgery*.

[75] Richoz O., Mavrakanas N., Pajic B., Hafezi F. (2013). Corneal collagen cross-linking for ectasia after LASIK and photorefractive keratectomy: long-term results. *Ophthalmology*.

[76] Vinciguerra P., Camesasca F. I., Albè E., Trazza S. (2010). Corneal collagen cross-linking for ectasia after excimer laser refractive surgery: 1-year results. *Journal of Refractive Surgery*.

[77] Hersh P. S., Greenstein S. A., Fry K. L. (2011). Corneal collagen crosslinking for keratoconus and corneal ectasia: one-year results. *Journal of Cataract & Refractive Surgery*.

[78] Cheema A. S., Mozayan A., Channa P. (2012). Corneal collagen crosslinking in refractive surgery. *Current Opinion in Ophthalmology*.

[79] Kanellopoulos A. J., Binder P. S. (2011). Management of corneal ectasia after LASIK with combined, same-day, topography-guided partial transepithelial PRK and collagen cross-linking: the Athens protocol. *Journal of Refractive Surgery*.

[80] Tae H. K., Lee D., Hyeon I. L. (2007). The safety of 250 *μ*m residual stromal bed in preventing keratectasia after laser in situ keratomileusis (LASIK). *Journal of Korean Medical Science*.

[81] Keay L., Edwards K., Naduvilath T. (2006). Microbial keratitis: predisposing factors and morbidity. *Ophthalmology*.

[82] Bourcier T., Thomas F., Borderie V., Chaumeil C., Laroche L. (2003). Bacterial keratitis: predisposing factors, clinical and microbiological review of 300 cases. *The British Journal of Ophthalmology*.

[83] Green M., Apel A., Stapleton F. (2008). Risk factors and causative organisms in microbial keratitis. *Cornea*.

[84] Saeed A., D'Arcy F., Stack J., Collum L. M., Power W., Beatty S. (2009). Risk factors, microbiological findings, and clinical outcomes in cases of microbial keratitis admitted to a tertiary referral center in Ireland. *Cornea*.

[85] Jeng B. H., Gritz D. C., Kumar A. B. (2010). Epidemiology of ulcerative keratitis in Northern California. *Archives of Ophthalmology*.

[86] Müller G., Goethe H., Herrmann R. (1972). Ship drinking water disinfection using UV irradiation. II. *Zentralblatt fur Bakteriologie, Parasitenkunde, Infektionskrankheiten und Hygiene. Erste Abteilung Originale. Reihe B: Hygiene, praventive Medizin*.

[87] Rau H. (1960). A transportable apparatus for UV irradiation in final disinfection of patient rooms. *Das Deutsche Gesundheitswesen*.

[88] Asano H., Lee C.-Y., Fox-Talbot K. (2007). Treatment with riboflavin and ultraviolet light prevents alloimmunization to platelet transfusions and cardiac transplants. *Transplantation*.

[89] Goodrich R. P. (2000). The use of riboflavin for the inactivation of pathogens in blood products. *Vox Sanguinis*.

[90] Spoerl E., Wollensak G., Seiler T. (2004). Increased resistance of crosslinked cornea against enzymatic digestion. *Current Eye Research*.

[91] Del Buey M. A., Cristóbal J. A., Casas P. (2012). Evaluation of in vitro efficacy of combined riboflavin and ultraviolet A for *Acanthamoeba* isolates. *American Journal of Ophthalmology*.

[92] Galperin G., Berra M., Tau J., Boscaro G., Zarate J., Berra A. (2012). Treatment of fungal keratitis from fusarium infection by corneal cross-linking. *Cornea*.

[93] Ferrari T. M., Leozappa M., Lorusso M., Epifani E., Ferrari L. M. (2009). *Escherichia coli* keratitis treated with ultraviolet A/riboflavin corneal cross-linking: a case report. *European Journal of Ophthalmology*.

[94] Li Z., Jhanji V., Tao X., Yu H., Chen W., Mu G. (2013). Riboflavin/ultravoilet light-mediated crosslinking for fungal keratitis. *The British Journal of Ophthalmology*.

[95] Müller L., Thiel M. A., Kipfer-Kauer A. I., Kaufmann C. (2012). Corneal cross-linking as supplementary treatment option in melting keratitis: a case series. *Klinische Monatsblatter fur Augenheilkunde*.

[96] Arance-Gil Á., Gutiérrez-Ortega Á. R., Villa-Collar C., Nieto-Bona A., Lopes-Ferreira D., González-Méijome J. M. (2014). Corneal cross-linking for *Acanthamoeba* keratitis in an orthokeratology patient after swimming in contaminated water. *Contact Lens & Anterior Eye*.

[97] Morén H., Malmsjö M., Mortensen J., Öhrström A. (2010). Riboflavin and ultraviolet a collagen crosslinking of the cornea for the treatment of keratitis. *Cornea*.

[98] Rama P., Di Matteo F., Matuska S., Paganoni G., Spinelli A. (2009). Acanthamoeba keratitis with perforation after corneal crosslinking and bandage contact lens use. *Journal of Cataract and Refractive Surgery*.

[99] Makdoumi K., Bäckman A., Mortensen J., Crafoord S. (2010). Evaluation of antibacterial efficacy of photo-activated riboflavin using ultraviolet light (UVA). *Graefe's Archive for Clinical and Experimental Ophthalmology*.

[100] Martins S. A. R., Combs J. C., Noguera G. (2008). Antimicrobial efficacy of riboflavin/UVA combination (365 nm) in vitro for bacterial and fungal isolates: a potential new treatment for infectious keratitis. *Investigative Ophthalmology & Visual Science*.

[101] Alio J. L., Abbouda A., Valle D. D., del Castillo J. M. B., Fernandez J. A. G. (2013). Corneal cross linking and infectious keratitis: a systematic review with a meta-analysis of reported cases. *Journal of Ophthalmic Inflammation and Infection*.

[102] McBrien N. A., Gentle A. (2003). Role of the sclera in the development and pathological complications of myopia. *Progress in Retinal and Eye Research*.

[103] Awetissow E. S. (1980). Data concerning a theory of origin of myopia. 3rd communication: the role of the sclera in the pathogenesis of progressive myopia. *Klinische Monatsblatter fur Augenheilkunde*.

[104] Nicoli S., Ferrari G., Quarta M. (2009). Porcine sclera as a model of human sclera for in vitro transport experiments: histology, SEM, and comparative permeability. *Molecular Vision*.

[105] Cruysberg L. P. J., Nuijts R. M. M. A., Geroski D. H., Gilbert J. A., Hendrikse F., Edelhauser H. F. (2005). The influence of intraocular pressure on the transscleral diffusion of high-molecular-weight compounds. *Investigative Ophthalmology & Visual Science*.

[106] Ambati J., Canakis C. S., Miller J. W. (2000). Diffusion of high molecular weight compounds through sclera. *Investigative Ophthalmology & Visual Science*.

[107] Wollensak G., Spoerl E. (2004). Collagen crosslinking of human and porcine sclera. *Journal of Cataract and Refractive Surgery*.

[108] Wollensak G., Iomdina E., Dittert D.-D., Salamatina O., Stoltenburg G. (2005). Cross-linking of scleral collagen in the rabbit using riboflavin and UVA. *Acta Ophthalmologica Scandinavica*.

[109] Zhang Y., Zou C., Liu L. (2013). Effect of irradiation time on riboflavin-ultraviolet-A collagen crosslinking in rabbit sclera. *Journal of Cataract & Refractive Surgery*.

[110] Olsen T. W., Edelhauser H. F., Lim J. I., Geroski D. H. (1995). Human scleral permeability: effects of age, cryotherapy, transscleral diode laser, and surgical thinning. *Investigative Ophthalmology and Visual Science*.

[111] Zhang Y., Li Z., Liu L., Han X., Zhao X., Mu G. (2014). Comparison of riboflavin/ultraviolet-A cross-linking in porcine, rabbit, and human sclera. *BioMed Research International*.

[112] Spoerl E., Seiler T. (1999). Techniques for stiffening the cornea. *Journal of Refractive Surgery*.

[113] Roberts J. E. (2001). Ocular photoxicity. *Journal of Photochemistry and Photobiology B: Biology*.

[114] Iseli H. P., Spoerl E., Wiedemann P., Krueger R. R., Seiler T. (2008). Efficacy and safety of blue-light scleral cross-linking. *Journal of Refractive Surgery*.

[115] Ahearne M., Yang Y., Then K. Y., Liu K.-K. (2008). Non-destructive mechanical characterisation of UVA/riboflavin crosslinked collagen hydrogels. *British Journal of Ophthalmology*.

