# Corrigendum to “A Review of Collagen Cross-Linking in Cornea and Sclera”

**DOI:** 10.1155/2016/7064093

**Published:** 2016-09-14

**Authors:** Xiao Zhang, Xiang-chen Tao, Jian Zhang, Zhi-wei Li, Yan-yun Xu, Yu-meng Wang, Chun-xiao Zhang, Guo-ying Mu

**Affiliations:** ^1^Department of Ophthalmology, Shandong Provincial Hospital Affiliated to Shandong University, Jinan, Shandong 250000, China; ^2^Zibo Eye Hospital, No. 33-6, Songlingxi Street, Zichuan, Zibo, Shandong 255100, China

In the article titled “A Review of Collagen Cross-Linking in Cornea and Sclera” [1], the first affiliation was incorrectly written as “Department of Ophthalmology, Shandong Provincial Hospital, Shandong University, Jinan, Shandong 250000, China,” while the correct address is “Department of Ophthalmology, Shandong Provincial Hospital Affiliated to Shandong University, Jinan, Shandong 250000, China.” The corrected affiliations are shown above.
